# Impact of Whole-Body Vibration Therapy in Elderly Populations: A Scoping Review

**DOI:** 10.7759/cureus.79296

**Published:** 2025-02-19

**Authors:** Gregory R Alfieri, Allison C Eaton, Kirk Dourvetakis, Melissa Rigueros, Trevor Creamean, Harvey N Mayrovitz

**Affiliations:** 1 Osteopathic Medicine, Nova Southeastern University Dr. Kiran C. Patel College of Osteopathic Medicine, Davie, USA; 2 Medical Education, Nova Southeastern University Dr. Kiran C. Patel College of Allopathic Medicine, Davie, USA

**Keywords:** arterial stiffness, balance, cardiovascular effects, geriatrics, muscle strength, posture, tremor, vibration health benefits, wbv, whole body vibration

## Abstract

The aging population faces neuromuscular stability, balance, and cardiovascular health challenges. This comes with a financial burden, morbidity, and reduced quality of life. Whole-body vibration (WBV) is a potential noninvasive therapy to address these challenges. This review aimed to determine and document the quality and extent of WBV therapy benefits in the elderly, as reported in the literature. OVID, EMBASE, and Web of Science were searched for peer-reviewed articles written in English between January 2008 and November 2024. Included were articles involving WBV effects on cardiovascular hemodynamics, neuromuscular stability, and sarcopenia in persons aged 50 and older. The initial search yielded 467 articles, and 37 were included for final review. The reported cardiovascular benefits of WBV included increased skin blood flow and improved markers of endothelial cell function. Mixed results exist for arterial stiffness metrics, and there was no significant impact of WBV on blood pressure. The most promising evidence relates to sarcopenia, where significant improvements were reported in muscle strength, performance, and functionality. Elderly persons with osteoarthritis had similar results, in addition to reduced pain and stiffness. Patients with Parkinson’s disease were reported to have improvements in tremor, rigidity, and postural stability. In contrast, stroke patients had mixed results in muscle activation but showed improvement in ankle joint proprioception. We conclude that although there is some evidence supporting the benefits of WBV on heart rate, muscle strength, function, and arterial stiffness, contradictory findings are reported. This points to the need for further research and a better definition of the optimal dosage of WBV, including its amplitude and frequency.

## Introduction and background

Background 

The average lifespan of humans is expected to continue to increase in the foreseeable future [1]. Specifically, by 2060, the number of individuals 65 years and older in the United States is expected to double to 96 million [2]. Cardiovascular and neuromuscular issues tend to increase with age and are more prevalent in the elderly. Arterial compliance decreases, while vessel thickness and aortic stiffness increase [2,3]. The costs associated with cardiovascular disease in those 65 and older will continue to increase in the future [1]. In addition, age-related loss of muscle mass and function (known as sarcopenia), decreased coordination of lower extremities, and diminished balance are of concern in the elderly, specifically for fall risk [4-7]. As a result, falls among the elderly are a significant issue, leading to health, social, and economic burdens [8]. With these burdens only expected to rise, it is imperative to seek an efficacious, feasible, and cost-effective treatment for cardiovascular, musculoskeletal, and balance issues in the elderly. Thus, whole-body vibration (WBV) therapy is potentially useful for addressing some of these issues. 

Whole-body vibration (WBV)

WBV is a therapeutic exercise modality that involves the exposure of the entire body to mechanical vibrations. The vibrations are typically generated by a platform device designed for this purpose. WBV operates on the principle of transmitting mechanical oscillations or vibrations to the body while an individual stands or sits on a vibrating platform or device [9]. Cardiovascular benefits of WBV have been reported to include an increase in skin blood flow (SBF) [10], pedal arterial velocity [11], improved endothelial cell function and nitric oxide production, and increased stroke volume and cardiac index [12,13]. Arterial stiffness was reported to decrease following WBV intervention [3]. Additionally, it has been reported that balance in the elderly has been shown to benefit from vibrational therapy [4,7,14]. Consensus currently exists that WBV improves physical function in older adults with sarcopenia [15,16], as well as muscle strength [17,18] and balance testing [19]. However, despite ample documentation of WBV's benefits, the consistency in the quantity, frequency, and duration necessary to obtain optimal results is lacking.

Goal 

The aging population faces neuromuscular stability, balance, and cardiovascular health challenges. These challenges include financial burden, morbidity, and reduced quality of life. WBV is a potential non-invasive therapy that may be useful in addressing some aspects of these challenges. Therefore, this scoping review aims to assess peer-reviewed research studies published on WBV therapy in elderly populations and characterize and describe its reported potential benefits on cardiovascular disease, sarcopenia, and balance in elderly populations.

## Review

Methods

*Overview* 

**Figure 1:**
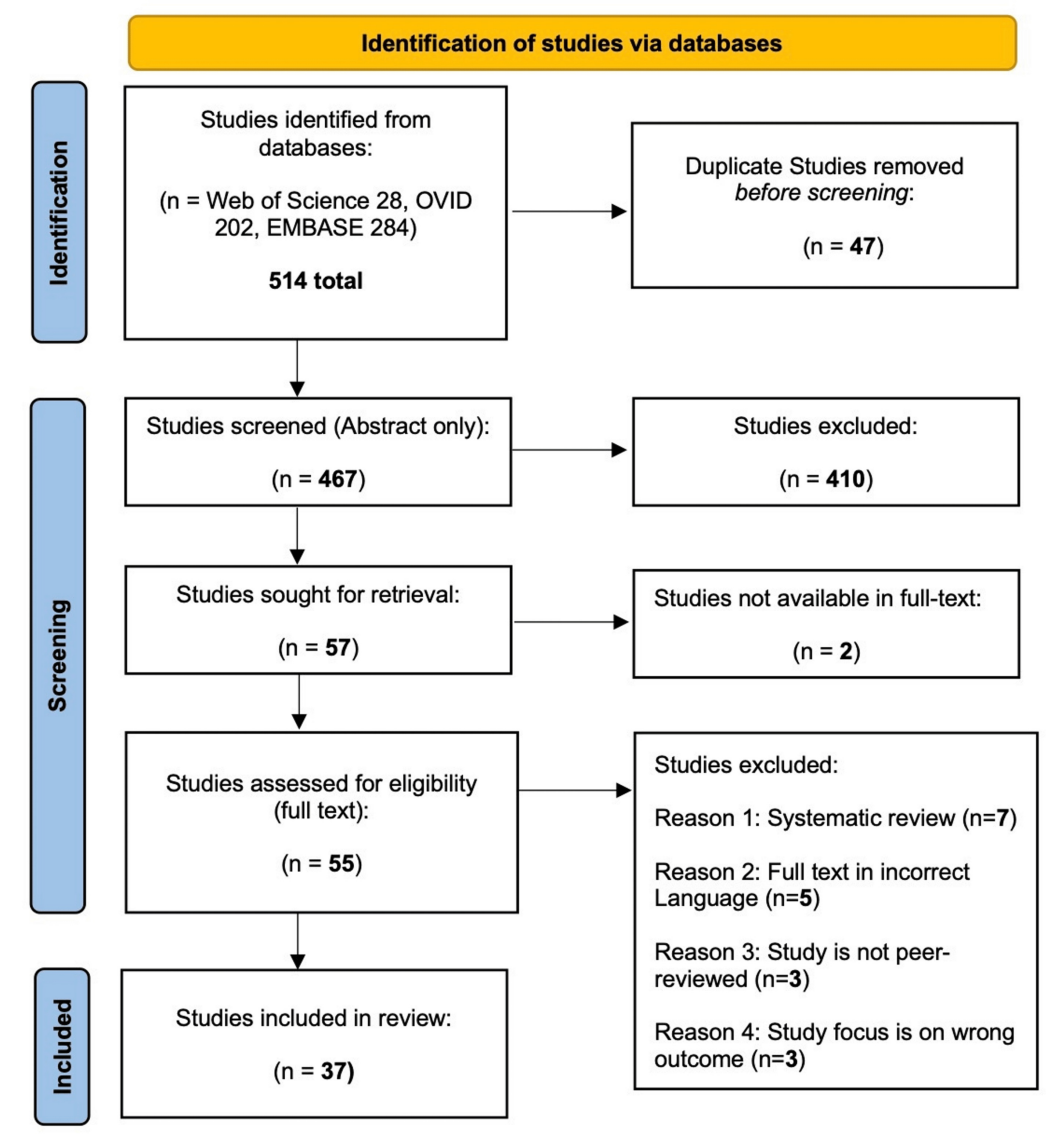
PRISMA flowchart of study selection This is a PRISMA flow diagram illustrating the selection process for studies included in the scoping review. The diagram details the number of records identified through database searching, the number of records screened, excluded, and assessed for eligibility, and the final number of studies included in the review. PRISMA, Preferred Reporting Items for Systematic Reviews and Meta-Analyses

This scoping review was performed in accordance with the Joanna Briggs Institute (JBI) methodology for scoping reviews [20]. A search of three databases was conducted for the literature on peer-reviewed articles written in English and published between 2008 and 2024 regarding WBV effects on cardiovascular hemodynamics, neuromuscular stability, and sarcopenia in persons aged 50 and older. The databases searched were OVID, EMBASE, and Web of Science. The search string included WBV training, geriatrics, hemodynamics, circulation, cardiovascular disease, and neuromuscular and musculoskeletal disease. Studies were excluded if they were not published within the desired timeframe, were not written in English, were not available in full text, used non-human subjects, did not focus on populations aged 50 years or older, or were systematic reviews, opinions, recommendations, dissertations, or books. The search results are presented in Figure 1 in a PRISMA-ScR (Preferred Reporting Items for Systematic Reviews and Meta-Analyses Extension for Scoping Reviews), following its appropriate guidelines [21]. After duplicate removal and screening for inclusion-exclusion criteria, the final literature review had 37 full-text articles. Most of the articles included were quantitative studies. Four studies related to muscle strength, proprioception, and balance in elderly patients; five studies were related to osteoarthritis (OA); 11 studies were related to sarcopenia; and eight studies were related to either stroke or Parkinson’s disease (PD) patients. Nine studies were related to cardiovascular and vascular health.

Study and Source of Evidence Selection

All identified citations were collated and uploaded into Endnote 21.0.1, released in 2023 (Clarivate Analytics, Philadelphia, PA, USA), and duplicates were removed. Independent reviewers screened titles and abstracts for assessment against the inclusion criteria for the review. The full text of selected citations was then assessed in detail against the inclusion criteria by two reviewers working independently. Reasons for excluding sources of evidence in the full text that do not meet the inclusion criteria are recorded and reported in Figure 1. Any disagreements between the reviewers at each stage of the selection process were resolved through discussion or with an additional reviewer.

Study Records - Data Management

Rayyan (Qatar Computing Research Institute, Ar-Rayyan, Qatar) and Excel software (Microsoft® Corp., Redmond, WA, USA) were used to manage records and data throughout this review. Rayyan is a web application that helps expedite the initial screening of abstracts and titles using a process of semi-automation, while incorporating a high level of usability [22].

Data Extraction

Two or more independent reviewers extracted data from papers included in the scoping review using Microsoft Excel as a data extraction tool. Data were organized using a modified version of a spreadsheet based on the Matrix Method [21].

Quality Evidence Assessment

The Quality Assessment Tool for Observational Cohort and Cross-Sectional Studies, developed by the National Heart, Lung, and Blood Institute, was used to assess the quality of the reviewed quantitative studies [23]. The Critical Appraisal Skills Programme appraisal tools were used to assess the quality of the qualitative and mixed-methods reviewed studies [24].

Search Strings Used

Table 1 shows the search string used for EMBASE, Table 2 shows the search string used for Web of Science, and Table 3 shows the search string used for OVID.

**Table 1:** EMBASE search string

#	Query	Results
#1	'whole body vibration'/exp OR 'whole body vibration training'/exp	2,381
#2	'whole body vibration':ab,ti,kw OR 'whole body vibration training':ab,ti,kw OR wbv:ab,ti,kw	3,843
#3	#1 OR #2	4,240
#4	'aged'/exp OR 'geriatrics'/exp OR 'geriatric disorder'/exp	4,191,021
#5	'aged':ab,ti,kw OR 'geriatric*':ab,ti,kw OR 'geriatric disorder*':ab,ti,kw	1,194,446
#6	#4 OR #5	4,942,283
#7	#3 AND #6	757
#8	'circulation'/exp OR 'blood flow'/exp OR 'cardiovascular disease'/de OR 'hemodynamic*'	1,183,424
#9	'circulation':ab,ti,kw OR 'blood flow':ab,ti,kw OR 'cardiovascular disease*':ab,ti,kw OR 'hemodynamic*':ab,ti,kw	1,087,333
#10	#8 OR #9	1,590,327
#11	'neuromuscular disease'/exp OR 'musculoskeletal disease'/exp	3,125,736
#12	'neuromuscular disease*':ab,ti,kw OR 'musculoskeletal disease*':ab,ti,kw	20,122
#13	#11 OR #12	3,128,272
#14	#7 AND #10	88
#15	#7 AND #13	267
#16	#14 OR #15	333
	Filters Applied: English, Publication years 2008-2024	284

**Table 2:** Web of Science search string

#	Query	Results
#1	TS=('whole body vibration*' OR 'whole body vibration training*')	6,122
#2	TS=('aged*' OR 'geriatric*' OR 'geriatric disorder*')	911,235
#3	#1 AND #2	285
#4	TS=('circulation*' OR 'blood flow*' OR 'cardiovascular disease*' OR 'hemodynamic*')	1,349,269
#5	TS=('neuromuscular disease*' OR 'musculoskeletal disease*')	45,693
#6	#3 AND #4	24
#7	#3 AND #5	11
#8	#6 OR #7	30
	Filters Applied: English, Publication years 2008-2024	28

**Table 3:** OVID search string

#	Query	Results
#1	(whole body vibration*' or 'whole body vibration').mp.	2,540
#2	circulation*'.mp.	297,924
#3	exp Blood Circulation/	219,421
#4	exp Hemodynamics/	644,698
#5	exp Cardiovascular Diseases/	2,309,299
#6	2 or 3 or 4 or 5	2,832,559
#7	exp Neuromuscular Diseases/	290,640
#8	exp Musculoskeletal Diseases/	995,600
#9	7 or 8	1,179,756
#10	('aged' or 'geriatric*' or 'geriatric disorder*').mp.	5,308,628
#11	1 and 10	675
#12	6 and 11	78
#13	9 and 11	163
#14	12 or 13	227
	Filters Applied: Publication years 2008-2024	202

Results

Impacts of WBV on Cardiovascular Parameters

Skin blood flow (SBF): SBF on the posterior calf was measured using laser Doppler imaging in 10 subjects before and after two types of 10-minute vibration stimulation. In one type, which the authors called active, the subject stood with one leg resting on a vibrating plate while the other leg was supported on a non-vibrating surface. In the other type of stimulation, the subject was in a horizontal position with the posterior calf in contact with the same vibrating plate. The measurements indicate no effect of the active form while standing on the plate. In contrast, an approximate doubling of the laser Doppler flow was observed with the passive vibration intervention measured after stopping the vibration [25,26]. SBF, also measured by a laser Doppler imaging method but on the foot dorsum, demonstrated an increase in SBF in a group of 30 subjects while they stood on a plate vibrating at various frequencies [26]. The largest increase measured after the exposure was about 57% and occurred when subjects experienced a vibration frequency of 25 Hz. However, in neither of these studies was the elevation in SBF evaluated beyond 10 minutes to determine if there was a sustained effect. SBF was also reported to be increased on the foot dorsum in a 10-person study of people with diabetes and/or peripheral neuropathy [10]. Based on their data, after five minutes of exposure to WBV, SBF increased on average by about 15%. However, there was no significant difference between WBV and SHAM standing effects on SBF beyond the immediate post-treatment effect.

Heart rate and blood pressure: In a study of patients with and without sarcopenia, the effect of WBV on hemodynamic responses to exercise was evaluated [27]. It was reported that squatting exercises performed while experiencing WBV at 40 Hz resulted in a greater heart rate than squatting without vibration [27]. A similar study conducted on 18 elderly subjects reported a WBV-related heart rate increase of only 7.5% [28]. In elderly patients with various cardiovascular diseases, squats done for 30 seconds while experiencing WBV at 30 Hz did not have a significantly elevated heart rate or blood pressure change [12]. A four-week program of one hour per day exposure to WBV at 20 Hz in patients with pulmonary artery hypertension (PAH) was reported to increase their six-minute walking distance [29]. However, data on heart rate or blood pressure changes were not provided. A study of nine octogenarians who underwent WBV did experience an elevation in heart rate and mean blood pressure during WBV. Still, this elevation did not differ from what they experienced in simulated WBV [30]. An important area in which no literature was found concerned the possible impact of WBV alone in lowering blood pressure in persons with hypertension. 

Vascular effects: To determine the effects of a single session of WBV on pulse wave velocity (PWV), nine elderly people were exposed to 10 minutes of simulated WBV and 10 minutes of WBV at a vibration frequency of 6 Hz, and central arterial pressures were determined [30]. From these pressure measurements, an augmentation index was determined and found to be increased due to the WBV. Since the augmentation index increases in the presence of an increased PWV, the inference from these findings is that WBV may have increased arterial stiffness. However, a longer study, utilizing a 12-week training program incorporating WBV in a group of 13 post-menopausal women with an average age of 64 years, reported a post-treatment decrease in the augmentation index [31]. A larger and longer study evaluated 70 people with an average age of 67.1 years before and after one year of WBV training, which resulted in an improvement in cardiopulmonary function [32]. In addition, one study found that the WBV treatment group saw a significant increase in reactive hyperemia peripheral arterial tonometry (RH PAT) index (a measure of endothelial function) [12].

Impacts of WBV on Sarcopenia

Regarding muscle strength, WBV therapeutic regimens were noted to improve strength with less workout time [32]. Specifically, there was strength improvement in hand grip, knee and hip extensors, isometric quadricep activity, and maximum leg extension and trunk flexion [17,33-35]. Similarly, another study reported a significant impact of WBV training on knee extension muscle strength and lower limb motor functions, such as jumping height, standing up, and walking tests in the elderly [36]. Regarding muscle performance and mobility, knee extensor performance reported improvements [16], while knee and hip extensors had increased mobility secondary to increased strength [33]. One study reported that shoulder-arm flexibility increased significantly and was the only study where increased skeletal muscle mass index saw significant improvement in the WBV group [17].

There was consistency in many functionality tests used in the quantitative studies addressing this matter, with one report of improvement in the timed up-and-go test, 10-minute timed walk, parallel walk test, and Barthel index score in the WBV arm [37]. Another study further supported this, finding significant improvements in the five repeated sit-to-stand tests, the eight-foot up-and-go test, and physical fitness standing on one foot [17]. One report saw significant improvement in the five sit-to-stand test and Tinetti test scores, but this improvement was noted in both the control and WBV groups [38]. There was one report with contradictory findings pertaining to the timed up-and-go test, where no statistically significant improvements were reported in WBV-exposed groups [26,38].

Impacts of WBV on OA

Five studies evaluated the effects of vibration therapy on muscle strength and function in older patients with OA, particularly in the knee. The experiments utilized assessments of functional strength and mobility, such as the timed up-and-go test, squat training, or other performance indexes, to compare changes from baseline after following a physical training routine incorporating vibration therapy in the experimental group. Compared with the control group or the intervention group that did not include vibration therapy, the vibration therapy group showed significantly improved muscle strength, proprioception, and functional capacity [3,39-41]. The use of vibration therapy in older populations with OA was found to be safe and useful in the conservative management of knee OA and may reduce pain, stiffness, and functional limitations in patients with knee OA [40,41].

*Impacts of WBV on Stroke and PD* 

Eight of the studies looked at the use of vibration therapy in those with previous strokes, as well as in PD. The studies regarding stroke evaluated a range of possible effects of WBV therapy, including effects on muscle strength and activation, balance, and cardiovascular stress [42-45]. Two of the stroke studies included were randomized controlled trials (RCTs), which indicated no significant differences between experimental and control groups following the use of WBV in conjunction with physical training. Metrics evaluated pre- and post-WBV training included muscle strength, lower limb muscle architecture, and balance [45,46]. On the other hand, an earlier study found WBV to be potentially beneficial for improving postural control, while another showed significantly greater muscle electromyography (EMG) activity observed with the use of WBV therapy in hemiparetic individuals with chronic stroke [43,44]. WBV therapy, in combination with physical exercise, induced significantly higher VO_2_, heart rate, and perceived exertion. Despite this increase in cardiovascular parameters, the use of WBV is suggested to be safe for use in those with chronic stroke [42]. Most recently, in 2024, a prospective cohort study was published evaluating the impact of WBV on ankle joint balance and proprioception. Stable stroke patients within the WBV group participated in rehabilitation programs, including exercise and physiotherapy, in addition to WBV. Compared to the rehabilitation-only group, the rehabilitation plus WBV cohort showed no significant improvement in ankle balance but did show significant improvement in ankle proprioception [47]. The studies regarding the use of WBV in those with PD assessed the effects of WBV on motor symptoms and postural stability of PD [48,49]. A crossover study assessed motor symptoms in 68 individuals with PD using the Unified Parkinson’s Disease Rating Scale (UPDRS) and found the use of WBV therapy showed improvement in tremor and rigidity [49]. Another study, using an RCT design, found randomized vibration, known as Stochastic Resonance Therapy (SRT), to have significantly enhanced postural stability in PD patients [26].

Impacts of WBV on Proprioception and Balance

A small experimental study that used 15 elderly women (age 63 ± 5 years) found that tests measuring functional balance before and after intervention with WBV treatment observed statistically altered sway measures in elderly women, suggesting improved proprioception and balance [50]. Two subsequent RCTs went on to show conflicting efficacy for WBV regarding balance and fall risk in elderly populations. The earlier study evaluated elderly people in nursing homes (10 centers, 159 total participants) and indicated no significant differences between the exercise group and the exercise plus WBV group when performing an exercise program of static and dynamic exercises over a six-week training period of three sessions per week. A subsequent RCT looking at WBV effects on intrinsic risk factors for falls in 42 women over 60 years old found that WBV therapy improves gait, balance, and functional strength [38,51]. Most recently, a study published in 2024 also reported non-significant improvements in lower limb function based on a short physical performance battery (SPPB), which includes a static balance test, a five-time chair-stand, and a 4 m normal gait speed test [52]. It has also been found that plantar vibrotactile perception increased significantly after WBV exposure, especially at higher frequencies, but there were no WBV-induced balance improvements [26].

Discussion 

Regarding SBF, findings showed significantly increased SBF when WBV was added to the experimental conditions. However, there was no significant difference between control and WBV arms beyond immediate post-treatment [10,25,26]. Although we present only three studies, there is consistency in the conclusion that WBV seems capable of increasing SBF. It has been documented that tissue perfusion and hydration are altered during the aging process, which can affect skin integrity and wound healing [53]. Thus, WBV can possibly serve as a modality to increase blood flow and, therefore, wound healing in areas where patients' skin has compromised circulation.

There was variability in the findings regarding the significance of WBV’s effect on heart rate. Some findings suggested a significant increase in heart rate in the WBV exercise group compared to the exercise-only group [27], while others demonstrated increased heart rate in the WBV experimental groups, yet without statistical significance [12,28]. It has been hypothesized that it is possible to estimate physical activity energy expenditure from heart rate [54]. Thus, an increased heart rate in elderly patients, without increasing resistance or session length, can potentially increase the intensity and caloric expenditure of the session without added musculoskeletal stress, thereby avoiding potential injury in a high-risk population. 

The findings concerning the effects of WBV on blood pressure are all in alignment: systolic and diastolic blood pressure do not change or decrease significantly between WBV and control groups [12,28,55]. With hypertension being a key health problem in the United States (the prevalence of hypertension in the USA in 2017-2018 was 49.64%) [56], patients could benefit from non-pharmacologic intervention. From our review, it seems that WBV may be of little use in lowering blood pressure in the elderly.

In all three studies regarding arterial hemodynamics, the findings were statistically significant, with two showing an improvement in arterial stiffness markers [31,55] and one showing large increases in stiffness measurements [30]. With cardiovascular disease as a leading cause of death in the United States in 2020 [57], these findings exhibit enough significant data to prompt further research into the mechanism by which WBV alters arterial stiffness. More studies need to be done to show if the net effect will be an increase or decrease in vessel stiffness. Both reported endothelial function studies showed improvement in function with the application of WBV [12,31]. With these findings, if endothelial function can be improved with WBV, this can serve as a potential modality for elderly patients with endothelial cell dysfunction or use the cell function improvement to augment blood flow to areas in need. The sole study addressing WBV in elderly PAH patients reported improved quality of life, exercise capacity, heart rate, and muscle strength in patients with the condition [30]. PAH is being increasingly diagnosed in elderly populations [58]; thus, with the improvement of all four parameters in this patient population, WBV can potentially be added as an adjunct to pharmacological regimens.

With respect to muscle strength, there was an improvement with less workout time in hand grip, knee and hip extensors, isometric quadriceps activity, and maximum leg extension and trunk flexion [12,17-21,23-35,59]. Knee extensor performance was improved [16]. Knee and hip extensor mobility increased secondary to increased strength [33], and shoulder-arm flexibility also increased significantly [17]. Overall, in an elderly population where the loss of mobility, strength, and muscle performance creates a reduced quality of life and can lead to fall-related injuries [60], WBV may help to improve these parameters across a wide array of muscle groups. If WBV can be added to a minimal exercise routine and improve the above-stated parameters, it can emerge as an opportunity to mitigate the effects of aging on the musculoskeletal system. The improvements were well documented among multiple studies, showing that one of WBV’s most legitimate applications is within the realm of addressing the deleterious effects of sarcopenia.

The benefits of WBV on functionality testing were widely reported. Improvements were found in the timed up-and-go test, 10-minute timed walk, parallel walk test, and Barthel index score in the WBV arm. Additionally, significant and non-significant improvements in the five repeated sit-to-stand tests, eight-foot up-and-go tests, and physical fitness standing on one foot were documented [17,39]. Contrarily, no statistically significant improvements were reported in the timed up-and-go test when exposed to WBV [27,39]. Although there were contradictory findings in functional testing, the net result of the evidence points to WBV as being effective in improving most functional testing. It has previously been shown that functional training may improve body composition and muscle strength in patients diagnosed with sarcopenia [61]; thus, if WBV can increase functional performance metrics in elderly populations, we can combat the deleterious effects of sarcopenia.

With regard to patients with OA, compared with the control group or the intervention group that did not include vibration therapy, the vibration therapy group showed significantly improved muscle strength, proprioception, and functional capacity [40-42,55]. WBV was found to be safe and useful in the conservative management of knee OA and may reduce pain, stiffness, and functional limitations in patients with knee OA [41,42]. OA is an age-related condition that causes joint pain, reduced mobility, and functional and social impairments, which can lead to depression and impose a significant social burden [62]. Our results regarding WBV in OA patients largely suggested positive benefits across many studies, showing that OA is a condition that can gain large improvements from WBV.

The results of this scoping review underscore the potential of WBV therapy as an intervention for addressing neuromuscular deficits in elderly individuals, particularly those affected by stroke and PD. Notably, improvements in cardiovascular parameters such as VO_2_ and heart rate, alongside enhanced postural control, are reported in the studies examining WBV therapy in stroke patients [43-46]. However, RCTs demonstrate inconsistent results, particularly regarding muscle strength and activation [46,47]. These discrepancies emphasize the need for optimizing treatment protocols and considering individual, non-standardized patient characteristics. Conversely, significant improvements in motor symptoms and postural stability are consistently observed following WBV intervention in PD patients [48,49]. Challenges such as heterogeneity in study design and measurement, in addition to limited long-term follow-up periods, warrant some caution in the adoption and interpretation of results [43-49]. 

This scoping review reveals mixed findings regarding the efficacy of WBV training in enhancing balance among older adults. No significant difference in balance and muscle performance was reported between those who underwent a six-week exercise program on a vibratory platform and those on a stationary surface. The two groups had no disparity in fall risks [38]. Moreover, another RCT revealed that WBV at 20 Hz was insufficient to improve balance among healthy older adults [26]. Acute effects of WBV may even negatively impact balance measures in elderly women [50]. This indicates adverse effects on balance, contradicting some previous studies suggesting improved balance with WBV, possibly due to differences in assessment methods and durations of exposure. Conversely, a more recent RCT demonstrated improved dynamic balance following a 12-week regimen of WBV therapy [51]. This suggests limited benefits of WBV training for balance improvement in this demographic.

Our findings' limitations primarily involve standardizing WBV therapeutic regimens. There is a lack of evidence that outlines the optimal frequency, quantity, and amplitude of vibrational therapy that elicits optimal results in each of the aforementioned pathologies. Another limitation is that there is not yet enough evidence for each condition, to create a rationale for healthcare providers to seek WBV over traditional and proven current therapies.

Future research regarding WBV's effects on cardiovascular health should be focused on defining the specific mechanism by which WBV improves arterial stiffness markers, endothelial cell function, and heart rate. More patients with hypertension and coronary artery disease must be recruited to see if the current findings in patients devoid of this condition can be replicated. In addition, studies with larger cohorts of PAH patients should implement WBV as an experimental arm, and study the specific hemodynamic and arterial markers in the pulmonary artery, as well as the mechanism of improvements in heart rate and exercise capacity in this population.

Although the benefit of WBV on sarcopenia and OA was well documented, future research should address different experimental groups with varied levels of exercise regimens and WBV exposure. This should be done to identify the optimal amount of exercise and WBV needed to decrease the effects of these conditions on functionality and muscle strength. WBV therapy, nonetheless, emerges as a promising and potentially cost-effective treatment modality for PD, while mostly contradictory evidence exists for the effects of WBV on stroke patients. Future standardized protocols and comprehensive longitudinal investigations are needed to elucidate clinical utility and long-term effects. Our findings concerning proprioception and balance may help explore the proper application of WBV training to improve balance and proprioception. Further studies are needed to establish guidelines on safe and effective vibration parameters to standardize the use of WBV. The authors acknowledged the need for larger samples and more extended studies to thoroughly assess the impact on fall risks.

## Conclusions

WBV seems to increase SBF, markers of endothelial cell function, and cardiopulmonary fitness in patients with PAH. There were mixed findings regarding WBV's effect on heart rate and augmentation index, and WBV does not seem to have a large impact on blood pressure. WBV shows improved postural control and mixed results on muscle activation and cardiovascular stress parameters in stroke patients. Perhaps the most substantial data exists to elucidate the benefits of WBV on sarcopenia and OA. Many sources report that WBV improves muscle strength, flexibility, mobility, and functionality. WBV significantly improves this patient population's muscle strength, proprioception, and functionality. It may also reduce pain, stiffness, and functional limitations in patients with OA of the knee. Mixed findings were reported for the effect of WBV on balance, as some studies reported significant increases, while others showed no additional benefit. Improvements in gait and functional strength, as well as improvement in fall risk factors, have been reported following WBV intervention. WBV improved tremor, rigidity, and postural stability in PD patients.

## References

[1] North BJ, Sinclair DA (2012). The intersection between aging and cardiovascular disease. Circ Res.

[2] Singam NS, Fine C, Fleg JL (2020). Cardiac changes associated with vascular aging. Clin Cardiol.

[3] Lai CL, Chen HY, Tseng SY (2014). Effect of whole-body vibration for 3 months on arterial stiffness in the middle-aged and elderly. Clin Interv Aging.

[4] Paolucci T, Pezzi L, La Verde R, Latessa PM, Bellomo RG, Saggini R (2021). The focal mechanical vibration for balance improvement in elderly - a systematic review. Clin Interv Aging.

[5] Bruyère O, Beaudart C, Ethgen O, Reginster JY, Locquet M (2019). The health economics burden of sarcopenia: a systematic review. Maturitas.

[6] Faulkner JA, Larkin LM, Claflin DR, Brooks SV (2007). Age-related changes in the structure and function of skeletal muscles. Clin Exp Pharmacol Physiol.

[7] Tseng SY, Hsu PS, Lai CL, Liao WC, Lee MC, Wang CH (2016). Effect of two frequencies of whole-body vibration training on balance and flexibility of the elderly: a randomized controlled trial. Am J Phys Med Rehabil.

[8] Alekna V, Stukas R, Tamulaitytė-Morozovienė I, Šurkienė G, Tamulaitienė M (2015). Self-reported consequences and healthcare costs of falls among elderly women. Medicina (Kaunas).

[9] Pel JJ, Bagheri J, van Dam LM, van den Berg-Emons HJ, Horemans HL, Stam HJ, van der Steen J (2009). Platform accelerations of three different whole-body vibration devices and the transmission of vertical vibrations to the lower limbs. Med Eng Phys.

[10] Johnson PK, Feland JB, Johnson AW, Mack GW, Mitchell UH (2014). Effect of whole body vibration on skin blood flow and nitric oxide production. J Diabetes Sci Technol.

[11] Robbins D, Yoganathan P, Goss-Sampson M (2014). The influence of whole body vibration on the central and peripheral cardiovascular system. Clin Physiol Funct Imaging.

[12] Aoyama A, Yamaoka-Tojo M, Obara S (2019). Acute effects of whole-body vibration training on endothelial function and cardiovascular response in elderly patients with cardiovascular disease. Int Heart J.

[13] Park SY, Son WM, Kwon OS (2015). Effects of whole body vibration training on body composition, skeletal muscle strength, and cardiovascular health. J Exerc Rehabil.

[14] Cheung WH, Mok HW, Qin L, Sze PC, Lee KM, Leung KS (2007). High-frequency whole-body vibration improves balancing ability in elderly women. Arch Phys Med Rehabil.

[15] Wu S, Ning HT, Xiao SM, Hu MY, Wu XY, Deng HW, Feng H (2020). Effects of vibration therapy on muscle mass, muscle strength and physical function in older adults with sarcopenia: a systematic review and meta-analysis. Eur Rev Aging Phys Act.

[16] Wei N, Pang MY, Ng SS, Ng GY (2017). Optimal frequency/time combination of whole body vibration training for developing physical performance of people with sarcopenia: a randomized controlled trial. Clin Rehabil.

[17] Chang SF, Lin PC, Yang RS, Yang RJ (2018). The preliminary effect of whole-body vibration intervention on improving the skeletal muscle mass index, physical fitness, and quality of life among older people with sarcopenia. BMC Geriatr.

[18] Jo NG, Kang SR, Ko MH, Yoon JY, Kim HS, Han KS, Kim GW (2021). Effectiveness of whole-body vibration training to Improve muscle strength and physical performance in older adults: prospective, single-blinded, randomized controlled trial. Healthcare (Basel).

[19] Dudoniene V, Sakaliene R, Svediene L, Kazlauskiene D, Szczegielniak J, Krutulyte G (2013). Impact of whole body vibration on balance improvement in elderly women. J Vibroeng.

[20] Peters MDJ GC, McInerney P, Munn Z, Tricco AC, Khalil H (2020). Scoping reviews. JBI Reviewer's Manual.

[21] Garrard J (2014). Health Sciences Literature Review Made Easy: The Matrix Method. https://samples.jbpub.com/9781284115192/9781284133950_FMxx_i_xvi.pdf.

[22] Ouzzani M, Hammady H, Fedorowicz Z, Elmagarmid A (2016). Rayyan-a web and mobile app for systematic reviews. Syst Rev.

[23] (2025). National heart, lung, and blood institute: study quality assessment tool for observational cohort and cross-sectional studies. https://www.nhlbi.nih.gov/health-topics/study-quality-assessment-tools.

[24] (2025). Checklist CASPCQ: critical appraisal skills programme CASP (qualitative) checklist. https://casp-uk.net/casp-tools-checklists/.

[25] Lohman EB 3rd, Sackiriyas KS, Bains GS (2012). A comparison of whole body vibration and moist heat on lower extremity skin temperature and skin blood flow in healthy older individuals. Med Sci Monit.

[26] Mahbub MH, Hase R, Yamaguchi N, Hiroshige K, Harada N, Bhuiyan AN, Tanabe T (2020). Acute effects of whole-body vibration on peripheral blood flow, vibrotactile perception and balance in older adults. Int J Environ Res Public Health.

[27] de Paula FA, Mendonça VA, Lage VK (2021). Immediate effects of whole-body vibration associated with squatting exercises on hemodynamic parameters in sarcopenic older people: a randomized controlled trial. Int J Environ Res Public Health.

[28] Avelar NC, Simão AP, Tossige-Gomes R (2011). Oxygen consumption and heart rate during repeated squatting exercises with or without whole-body vibration in the elderly. J Strength Cond Res.

[29] Gerhardt F, Dumitrescu D, Gärtner C (2017). Oscillatory whole-body vibration improves exercise capacity and physical performance in pulmonary arterial hypertension: a randomised clinical study. Heart.

[30] Abdolhosseini P, Lark S, Wadsworth D, Stoner L (2019). The effects of acute bouts of whole body vibration on central hemodynamics in frail older adults: a pilot study. Phys Occup Ther Geriatr.

[31] Jaime SJ, Maharaj A, Alvarez-Alvarado S, Figueroa A (2019). Impact of low-intensity resistance and whole-body vibration training on aortic hemodynamics and vascular function in postmenopausal women. Hypertens Res.

[32] Bogaerts AC, Delecluse C, Claessens AL, Troosters T, Boonen S, Verschueren SM (2009). Effects of whole body vibration training on cardiorespiratory fitness and muscle strength in older individuals (a 1-year randomised controlled trial). Age Ageing.

[33] Machado A, García-López D, González-Gallego J, Garatachea N (2010). Whole-body vibration training increases muscle strength and mass in older women: a randomized-controlled trial. Scand J Med Sci Sports.

[34] Simão AP, Mendonça VA, Avelar NC (2019). Whole body vibration training on muscle strength and brain-derived neurotrophic factor levels in elderly woman with knee osteoarthritis: a randomized clinical trial study. Front Physiol.

[35] von Stengel S, Kemmler W, Engelke K, Kalender WA (2012). Effect of whole-body vibration on neuromuscular performance and body composition for females 65 years and older: a randomized-controlled trial. Scand J Med Sci Sports.

[36] Hu J, Wang Y, Ji X, Zhang Y, Li K, Huang F (2024). Non-pharmacological strategies for managing sarcopenia in chronic diseases. Clin Interv Aging.

[37] Wadsworth D, Lark S (2020). Effects of whole-body vibration training on the physical function of the frail elderly: an open, randomized controlled trial. Arch Phys Med Rehabil.

[38] Sitjà-Rabert M, Martínez-Zapata MJ, Fort Vanmeerhaeghe A, Rey Abella F, Romero-Rodríguez D, Bonfill X (2015). Effects of a whole body vibration (WBV) exercise intervention for institutionalized older people: a randomized, multicentre, parallel, clinical trial. J Am Med Dir Assoc.

[39] Trans T, Aaboe J, Henriksen M, Christensen R, Bliddal H, Lund H (2009). Effect of whole body vibration exercise on muscle strength and proprioception in females with knee osteoarthritis. Knee.

[40] Salmon JR, Roper JA, Tillman MD (2012). Does acute whole-body vibration training improve the physical performance of people with knee osteoarthritis?. J Strength Cond Res.

[41] Rabini A, de Sire A, Marzetti E (2015). Effects of focal muscle vibration on physical functioning in patients with knee osteoarthritis: a randomized controlled trial. Eur J Phys Rehabil Med.

[42] Liao LR, Ng GY, Jones AY, Pang MY (2015). Cardiovascular stress induced by whole-body vibration exercise in individuals with chronic stroke. Phys Ther.

[43] Huang M, Pang MY (2019). Muscle activity and vibration transmissibility during whole-body vibration in chronic stroke. Scand J Med Sci Sports.

[44] van Nes IJ, Geurts AC, Hendricks HT, Duysens J (2004). Short-term effects of whole-body vibration on postural control in unilateral chronic stroke patients: preliminary evidence. Am J Phys Med Rehabil.

[45] Marín PJ, Ferrero CM, Menéndez H, Martín J, Herrero AJ (2013). Effects of whole-body vibration on muscle architecture, muscle strength, and balance in stroke patients: a randomized controlled trial. Am J Phys Med Rehabil.

[46] Brogårdh C, Flansbjer UB, Lexell J (2012). No specific effect of whole-body vibration training in chronic stroke: a double-blind randomized controlled study. Arch Phys Med Rehabil.

[47] Xu P, Song J, Fan W (2024). Impact of whole-body vibration training on ankle joint proprioception and balance in stroke patients: a prospective cohort study. BMC Musculoskelet Disord.

[48] Kaut O, Brenig D, Marek M, Allert N, Wüllner U (2016). Postural stability in Parkinson's disease patients is improved after stochastic resonance therapy. Parkinsons Dis.

[49] Haas CT, Turbanski S, Kessler K, Schmidtbleicher D (2006). The effects of random whole-body-vibration on motor symptoms in Parkinson's disease. NeuroRehabilitation.

[50] Dabbs NC, MacDonald CJ, Chander H, Lamont HS, Garner JC (2014). The effect of whole-body vibration on balance in elderly women. Med Sportiva.

[51] Nawrat-Szołtysik A, Sieradzka M, Nowacka-Chmielewska M, Piejko L, Duda J, Brachman A, Polak A (2022). Effect of whole-body vibration training on selected intrinsic risk factors in women aged 60+ at fall risk: a randomized controlled trial. Int J Environ Res Public Health.

[52] Sievänen H, Piirtola M, Tokola K (2024). Effect of 10-week whole-body vibration training on falls and physical performance in older adults: a blinded, randomized, controlled clinical trial with 1-year follow-up. Int J Environ Res Public Health.

[53] Cowan L, Broderick V, Alderden JG (2020). Pressure injury prevention considerations for older adults. Crit Care Nurs Clin North Am.

[54] Keytel LR, Goedecke JH, Noakes TD, Hiiloskorpi H, Laukkanen R, van der Merwe L, Lambert EV (2005). Prediction of energy expenditure from heart rate monitoring during submaximal exercise. J Sports Sci.

[55] Lai CL, Chen HY, Tseng SY, Liao WC, Liu BT, Lee MC, Chen HS (2014). Effect of whole-body vibration for 3 months on arterial stiffness in the middle-aged and elderly. Clin Interv Aging.

[56] Chobufo MD, Gayam V, Soluny J (2020). Prevalence and control rates of hypertension in the USA: 2017-2018. Int J Cardiol Hypertens.

[57] Curtin SC, Tejada-Vera B, Bastian BA (2023). Deaths: leading causes for 2020. Natl Vital Stat Rep.

[58] Rothbard N, Agrawal A, Fischer C, Talwar A, Sahni S (2020). Pulmonary arterial hypertension in the elderly: clinical perspectives. Cardiol J.

[59] Shruthi R, Jyothi R, Pundarikaksha HP, Nagesh GN, Tushar TJ (2016). A study of medication compliance in geriatric patients with chronic illnesses at a tertiary care hospital. J Clin Diagn Res.

[60] Larsson L, Degens H, Li M (2019). Sarcopenia: aging-related loss of muscle mass and function. Physiol Rev.

[61] Mile M, Balogh L, Papp G (2021). Effects of functional training on sarcopenia in elderly women in the presence or absence of ACE inhibitors. Int J Environ Res Public Health.

[62] Naselli F, Bellavia D, Costa V, De Luca A, Raimondi L, Giavaresi G, Caradonna F (2023). Osteoarthritis in the elderly population: preclinical evidence of nutrigenomic activities of flavonoids. Nutrients.

